# Supplementary material for: Modulation of Visual Responses and Ocular Dominance by Contralateral Inhibitory Activation in the Mouse Visual Cortex
Source: Int J Mol Sci. 2023 Mar 17;24(6):5750. doi: 10.3390/ijms24065750 (PMC10058019; doi:10.3390/ijms24065750)
Supplement: Supplementary file 1 [file ijms-24-05750-s001.zip › ijms-2257630-supplementary.pdf]

Figure	Sample size (n)	Statistical test	P value
Fig. 1i	VGAT, n = 5<br>PV, n = 5<br>SST, n = 4	one-way ANOVA	Near,<br>F(2,11) = 7.231, p= 0.0099<br>Turcky's multiple comparisons test:<br>VGAT vs. PV, p = 0.0136<br>VGAT vs. SST, p = 0.9688<br>PV vs. SST, p = 0.0288<br>Far,<br>F(2,11) = 0.7875, p= 0.4790<br>Turcky's multiple comparisons test:<br>VGAT vs. PV, p = 0.4478<br>VGAT vs. SST, p = 0.8406<br>PV vs. SST, p = 0.8140
Fig. 1j	VGAT, n = 5<br>PV, n = 5<br>SST, n = 4	one-way ANOVA	Near,<br>F(2,11) = 8.127, p= 0.0068<br>Turcky's multiple comparisons test:<br>VGAT vs. PV, p = 0.0064<br>VGAT vs. SST, p = 0.6341<br>PV vs. SST, p = 0.0463<br>Far,<br>F(2,11) = 9.238, p= 0.0044<br>Turcky's multiple comparisons test:<br>VGAT vs. PV, p = 0.0055<br>VGAT vs. SST, p = 0.0184<br>PV vs. SST, p = 0.8800
Fig. 3c	n = 5	two-way RM ANOVA	Ipsi:<br>Intensity, F(5,20) = 1.935, p = 0.1332<br>Laser, F(1,4) = 3.750, p = 0.1249<br>Intensity x laser,<br>F(5,20) = 0.6586, p = 0.6588<br>Cont:<br>Intensity, F(5,20) = 3.005, p = 0.0350<br>Laser, F(1,4) = 0.0271, p = 0.8773<br>Intensity x laser,<br>F(5,20) = 1.002, p = 0.4420<br>Mono:<br>Intensity, F(5,20) = 0.7633, p = 0.5869<br>Laser, F(1,4) = 0.1817, p = 0.6918<br>Intensity x laser,<br>F(5,20) = 0.5713, p = 0.7211
Fig. 3d	n = 8	two-way RM ANOVA	Ipsi:<br>Intensity, F(5,35) = 17.53, p < 0.0001<br>Laser, F(1,7) = 71.03, p < 0.0001<br>Intensity x laser,<br>F(5,35) = 18.09, p < 0.0001<br>Cont:<br>Intensity, F(5,35) = 5.198, p = 0.0011<br>Laser, F(1,7) = 41.89, p = 0.0003<br>Intensity x laser,<br>F(5,35) = 6.722, p = 0.0002

			Mono:<br>Intensity, F(5,35) = 3.351, p = 0.0141<br>Laser, F(1,7) = 109.8, p < 0.0001<br>Intensity x laser,<br>F(5,35) = 5.932, p = 0.0005
Fig. 3e	n = 9	two-way RM ANOVA	Ipsi:<br>Intensity, F(5,40) = 2.910, p = 0.0248<br>Laser, F(1,8) = 29.31, p = 0.0006<br>Intensity x laser,<br>F(5,40) = 15.29, p < 0.0001<br>Cont:<br>Intensity, F(5,40) = 1.101, p = 0.3751<br>Laser, F(1,8) = 64.49, p < 0.0001<br>Intensity x laser,<br>F(5,40) = 6.553, p = 0.0002<br>Mono:<br>Intensity, F(5,40) = 0.3742, p = 0.8622<br>Laser, F(1,8) = 19.47, p = 0.0022<br>Intensity x laser,<br>F(5,40) = 1.536, p = 0.2005
Fig. 3f	n = 4	two-way RM ANOVA	Ipsi:<br>Intensity, F(5,15) = 3.855, p = 0.0191<br>Laser, F(1,3) = 451.0, p = 0.0002<br>Intensity x laser,<br>F(5,15) = 6.415, p = 0.0022<br>Cont:<br>Intensity, F(5,15) = 3.156, p = 0.0383<br>Laser, F(1,3) = 95.32, p = 0.0023<br>Intensity x laser,<br>F(5,15) = 6.670, p = 0.0019<br>Mono:<br>Intensity, F(5,15) = 3.002, p = 0.0450<br>Laser, F(1,3) = 42.01, p = 0.0075<br>Intensity x laser,<br>F(5,15) = 6.357, p = 0.0023
Fig. 4c	n = 4	two-way RM ANOVA	Ipsi:<br>Intensity, F(5,15) = 0.6802, p = 0.6453<br>Laser, F(1,3) = 0.4660, p = 0.5438<br>Intensity x laser,<br>F(5,15) = 1.831, p = 0.1671<br>Cont:<br>Intensity, F(5,15) = 1.623, p = 0.2142<br>Laser, F(1,3) = 0.7631, p = 0.4467<br>Intensity x laser,<br>F(5,15) = 0.8962, p = 0.5105<br>Mono:<br>Intensity, F(5,15) = 0.4859, p = 0.8006<br>Laser, F(1,3) = 0.2675, p = 0.6407

			Intensity x laser,<br>F(5,15) = 0.4055, p = 0.8375
Fig. 4d	n = 9	two-way RM ANOVA	Ipsi:<br>Intensity, F(5,40) = 1.155, p = 0.3479<br>Laser, F(1,8) = 33.18, p = 0.0004<br>Intensity x laser,<br>F(5,40) = 2.636, p = 0.0376<br>Cont:<br>Intensity, F(5,40) = 0.5663, p = 0.7252<br>Laser, F(1,8) = 18.13, p = 0.0028<br>Intensity x laser,<br>F(5,40) = 1.171, p = 0.3405<br>Mono:<br>Intensity, F(5,40) = 0.9449, p = 0.4628<br>Laser, F(1,8) = 3.528, p = 0.0972<br>Intensity x laser,<br>F(5,40) = 0.7674, p = 0.5788
Fig. 5a	WT,<br>n= 5<br>VGAT-ChR2,<br>n = 8<br>PV-ChR2,<br>n = 9<br>SST-ChR2,<br>n = 4	two-way RM ANOVA	WT:<br>Intensity, F(5,20) = 0.8214, p = 0.5489<br>Laser, F(1,4) = 2.154, p = 0.2161<br>Intensity x laser,<br>F(5,20) = 0.4149, p = 0.8328<br>VGAT:<br>Intensity, F(5,35) = 8.678, p < 0.0001<br>Laser, F(1,7) = 36.63, p = 0.0004<br>Intensity x laser,<br>F(5,35) = 9.294, p < 0.0001<br>PV:<br>Intensity, F(5,40) = 2.770, p = 0.0306<br>Laser, F(1,8) = 18.06, p = 0.0028<br>Intensity x laser,<br>F(5,40) = 5.765, p = 0.0004<br>SST:<br>Intensity, F(5,15) = 3.404, p = 0.0297<br>Laser, F(1,3) = 20.02, p = 0.0208<br>Intensity x laser,<br>F(5,15) = 1.919, p = 0.1507
Fig. 5b	EYFP, n = 4<br>ChR2, n = 9	two-way RM ANOVA	EYFP:<br>Intensity, F(5,15) = 0.8877, p = 0.5134<br>Laser, F(1,3) = 0.4197, p = 0.5633<br>Intensity x laser,<br>F(5,15) = 1.741, p = 0.1860<br>ChR2:<br>Intensity, F(5,40) = 0.8546, p = 0.5198<br>Laser, F(1,8) = 14.49, p = 0.0052<br>Intensity x laser,<br>F(5,40) = 1.116, p = 0.3693

Fig. 6c	n = 4	Paired t-test	ipsi, p = 0.5773,<br>cont, p = 0.2240,<br>ODI, p = 0.4934
Fig. 6d	n = 5	Paired t-test	ipsi, p = 0.0131,<br>cont, p = 0.6121,<br>ODI, p = 0.0398

Table S1. Summary of statistical analysis.
